# Identification of Broad-Genotype HPV L2 Neutralization Site for Pan-HPV Vaccine Development by a Cross-Neutralizing Antibody

**DOI:** 10.1371/journal.pone.0123944

**Published:** 2015-04-23

**Authors:** Daning Wang, Zhihai Li, Jieqiong Xiao, Junqi Wang, Li Zhang, Yajing Liu, Fei Fan, Lu Xin, Minxi Wei, Zhibo Kong, Hai Yu, Ying Gu, Jun Zhang, Shaowei Li, Ningshao Xia

**Affiliations:** 1 State Key Laboratory of Molecular Vaccinology and Molecular Diagnostics, School of Public Health, Xiamen University, Xiamen, 361005, China; 2 National Institute of Diagnostics and Vaccine Development in Infectious Disease, School of Life Sciences, Xiamen University, Xiamen, 361005, China; International Centre for Genetic Engineering and Biotechnology, ITALY

## Abstract

Human Papillomavirus (HPV), a non-enveloped, double-stranded DNA virus, is responsible for 5% of human cancers. The HPV capsid consists of major and minor structural proteins, L1 and L2. L1 proteins form an icosahedral shell with building blocks of the pentameric capsomere, and one L2 molecule extends outward from the central hole of the capsid. Thus, L2 is concealed within L1 and only becomes exposed when the capsid interacts with host cells. The low antigenic variation of L2 means that this protein could offer a target for the development of a pan-HPV vaccine. Toward this goal, here we describe an anti-L2 monoclonal antibody, 14H6, which broadly neutralizes at least 11 types of HPV, covering types 6, 11, 16, 18, 31, 33, 35, 45, 52, 58 and 59, in pseudovirion—based cell neutralization assay. The mAb 14H6 recognizes a minimal linear epitope located on amino acids 21 to 30 of the L2 protein. Alanine scanning mutagenesis and sequence alignment identified several conserved residues (Cys22, Lys23, Thr27, Cys28 and Pro29) that are involved in the 14H6 binding with L2. The epitope was grafted to several scaffolding proteins, including HPV16 L1 virus-like particles, HBV 149 core antigen and CRM197. The resultant chimeric constructs were expressed in *Escherichia coli* and purified with high efficiency. Immunization with these pan-HPV vaccine candidates elicited high titers of the L2-specific antibody in mice and conferred robust (3-log) titers of cross-genotype neutralization, including against HPV11, 16, 18, 45, 52, 58 and 59. These findings will help in the development of an L2-based, pan-HPV vaccine.

## Introduction

With about 527,000 individuals affected every year, human papillomavirus (HPV) is the leading cause of cervical cancer and one of the most common causes of sexually transmitted disease [1,2]. Over 170 HPV genotypes have been identified [3], with chronic infection often resulting in skin lesions such as anogenital warts or, in the most serious cases malignant tumor formation [4]. Among the approximately 15 oncogenic types, HPV types 16 and 18 are responsible for 70% of cervical cancer and 5% of worldwide cancer [5–7]. There are currently two HPV prophylactic vaccines on the market: Gardasil by Merck & Co. (West Point, PA) and Cervarix by GlaxoSmithKline (Brentford, UK). Both vaccines are designed based on the *in vitro* self-assembly of L1 to form virus-like particles (VLPs) [8,9]. VLPs resemble the native viral capsid and can induce the high-titer formation of protective antibodies [8]; however, such immunities are almost always type-restricted to HPV16 and -18 (Gardasil and Cervarix, respectively), which accounts for approximately 70% protection from cervical cancer [10,11]. Patients thus remain unprotected against the other 30% of cervical cancer, caused by HPV31, 33, 35, 39, 44, 45, 51, 52, 58, 59, 66, 68. Minor cross-protection for these two vaccines has been found [4,12], with the genotype range for Gardasil extended to nine-valent HPV, which accounts for up to 87% cervical cancer; the phase 3 clinical trial for these tests are ongoing.

The HPV capsid consists of major and minor structural proteins, L1 and L2. The minor capsid antigen L2 plays a key role in immune escape and in the derivation of emerging genotypes [13]. This is likely due to its low antigenic variation. In recent years, much emphasis has thus been directed toward L2 for the development of a pan-HPV vaccine [14–17]. L2 functions in both virion assembly and the infection process, but it is concealed inside the L1 pentamer [13] and its neutralization epitopes are thought to be transiently exposed via furin cleavage during the infection process; this is guided by the interaction between L1 and a heparan sulfate proteoglycan (HSPG), which triggers a surface conformational change of the viral capsid [18,19]. Consequently, natural infection or immunization against the HPV L1/L2 pseudovirions (PsVs) would therefore produce predominantly L1 antibodies with little or no L2 antibodies. The alleviated selective pressure for antigenic variance, in turn, leads to a greater structural conservation. Through the analysis of RG1—an antibody that cross-neutralizes HPV 16 and 18—Gambhira et al. revealed that amino acids (aa) 17–36 of L2, which are exposed after furin cleavage, appear to be highly conserved among the different genotypes [20]. Similarly, study of K4L2 and K18L2, two cross-neutralizing antibodies, showed target sites at aa 20–38 of L2 [21].

Although cross-protective immunity is possible through L2 vaccination with either the full-length protein or an oligopeptide, the cross-neutralization range is still narrow and the antibody titers are low. Recent strategies have employed heterologous, modified L2 oligopeptide(s) that are created using repeats or grafted to various scaffold antigens. Indeed, VLPs from MS2 bacteriophage, PP7 bacteriophage, HPV, and adeno-associated virus-VP3, as well as flagellin and modified IgG Fc, were able to dramatically enhance the titer of highly cross-reactive and cross-protective antibodies against both vaccine and non-vaccine HPV genotypes [14,15,22–24]. Furthermore, a diphtheria toxin (DT) mutant, CRM197, has been extensively used as an intramolecular adjuvant to enhance the immunogenicity of certain antigens, including polysaccharides and haptens [25–27]. CRM197 is an A-subfragment mutant (Gly-52 to Glu) of DT and is currently used in Meningitec and Menveo for meningitis to improve immunogenicity [28,29]. For other VLPs, CRM197 may act as an internal adjuvant to improve T-cell immunity. DT contains three domains—receptor-binding domain R (amino acids 385–535), transmembrane domain T (amino acids 201–384) and catalytic domain A (amino acids 1–191) [30,31]—and we posit that deletion of domain R or domains R and T would result in the truncated CRM389 (aa 1–389) and CRMA (aa 1–191), respectively, which could also act as scaffold antigens.

In this study, we screened for HPV L2 broad-spectrum neutralizing antibodies from mice immunized with recombinant HPV16-L2 protein. The mAb 14H6, recognizing a linear epitope, was found to neutralize a remarkably broad range of HPV genotypes including HPV6, 11, 16, 18, 31, 33, 45, 52, 58 and 59 PsVs. Further, we grafted the peptide to various scaffold antigens, including CRM197, CRM389, CRMA, HBV core antigen, and HPV16 L1 VLPs, and measured the immunogenicity of L2 broad-spectrum epitope in mice. Overall, we demonstrate that 14H6 identifies a minimally conserved, broad-genotype neutralization site on HPV L2 and its corresponding epitope is immunogenic in mice with the aid of the scaffold proteins. These findings provide insight for an L2-based pan-HPV vaccine design and offer a promising vaccine candidate for a broad-spectrum HPV vaccine.

## Results

### 14H6 mAb, a broad, cross-neutralizing L2 antibody

**Fig 1:**
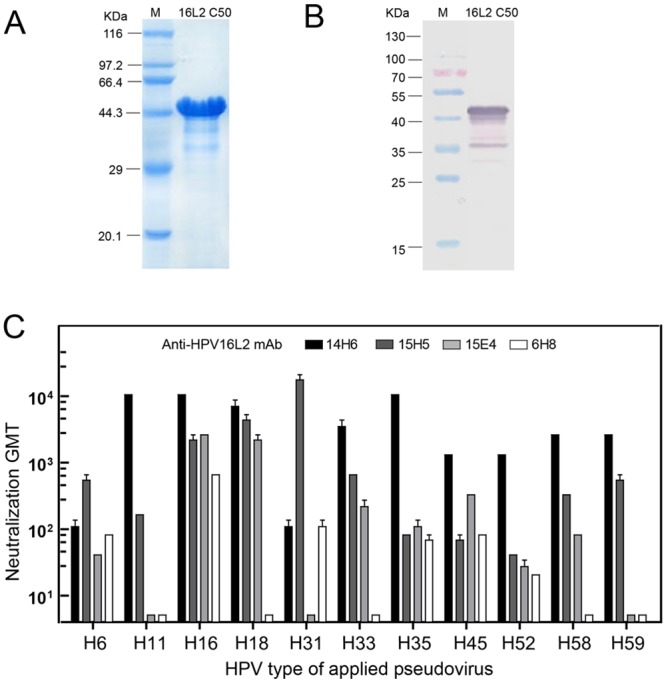
Screening for L2 neutralizing monoclonal antibodies (mAbs) using HPV16L2 C50 antigen. **(A)** SDS-PAGE analysis of purified HPV16L2 C50 antigen. **(B)** Western blotting of HPV16L2 C50 with a mouse anti-HPV16L2 antiserum. **(C)** Neutralization geometric mean titers (GMTs) of mAbs 14H6, 15H5, 15E4, 6H8 mAbs were plotted on the log-scaled *y*-axis against various genotypes of HPV-PsVs on the *x*-axis. The bar graph was prepared using Prism GraphPad 5.0. All mAb samples were adjusted to an initial concentration of 1.0 mg/ml prior to two-fold serial dilutions. Flow cytometry was used to detect the number of HPV-pseudovirus-infected enhanced green fluorescent protein (EGFP)-expressing cells. The collected data were processed to calculate the neutralizing titer and IC_50_ for each mAb, as shown in Table 1.

Previous research has shown that the HPV-L2 protein contains numerous, broad, cross-neutralizing epitopes [20,21]. To unravel more of these L2 epitopes, we immunized Balb/c mice with recombinant HPV16 L2 C50 protein, derived using the *Escherichia coli* expression system, and raised monoclonal antibodies by L2-based ELISA and a pseudovirion-based cell neutralization assay. The immunogen HPV16L2 C50 protein was resolved using SDS-PAGE (Fig 1A), and reacted with immune serum of HPV16L2 (Fig 1B). Eventually, 17 hybridomas secreting anti-HPV16-L2 monoclonal antibodies (mAbs) were deemed positive and, of those, 4 mAbs—14H6, 15H5, 15E4, and 6H8—were found to confer broad genotype neutralization in the cell model assay (Fig 1C). The properties for these mAbs are summarized in Table 1. Remarkably, mAb 14H6 presents with a broad, cross-inhibitory capacity against the infection of HPV6, 11, 16, 18, 31, 33, 35, 45, 52, 58 and 59 PsVs, with a relatively lower EC50 value, ranging from 0.1 to 6.25 μg/ml, and displaying a preference over the other mAbs.

**Table 1:** Characteristics of the HPV16-L2 mAbs.

				50% Inhibitory concentration (μg/mL) of mAbs against HPV-PsV infection										
mAbs	Epitope recognized	Isotype^b^	Titer^c^	6	11	16	18	31	33	35	45	52	58	59
14H6	L^a^	IgG1	10^8^	0.78	0.10	0.10	0.15	6.25	0.39	0.10	0.10	0.58	0.58	0.47
15E5	L	IgG1	10^7^	1.61	6.25	0.47	0.23	0.06	1.61	12.50	12.50	25.00	3.13	1.87
15E4	L	IgG1	10^6^	25.00	>50	0.39	0.47	>50	4.69	9.37	3.13	37.36	12.50	>50
6H8	L	IgG1	10^7^	12.50	>50	1.56	>50	9.37	>50	14.93	12.50	50.00	>50	>50

HPV, Human Papillomavirus; mAb, monoclonal antibody; PsVs, pseudovirus.

^a^ L, linear epitope, confirmed by reduced SDS-PAGE and western blotting.

^b^ antibody isotype, classified by SBA Clonotyping System-HRP (SouthernBiotech, USA).

^c^ Total IgG antibody titer, measured by L2-based ELISA.

### Epitope mapping and key interaction residues for 14H6

**Fig 2:**
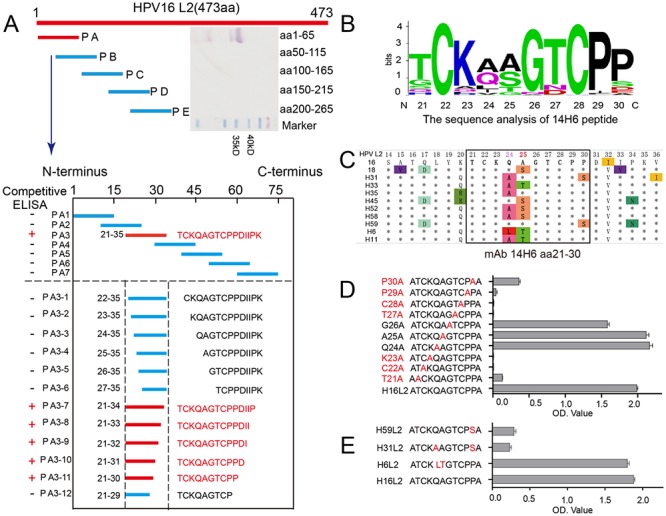
Delineation of HPV16-L2 14H6 epitope. **(A)** Localization of the linear epitope of mAb 14H6 by L2 fragments and overlapping peptide scanning. First, mAb 14H6 was shown to only react with peptide A (PA) construct (aa 1–65) in western blotting, indicating that its corresponding epitope was located in the N-terminal fragment of HPV16-L2 (upper panel). Next, we synthesized overlapping peptides spanning aa 1–75, and this narrowed down the epitope recognition to PA3 peptide (aa 21–35) (middle panel). Finally, both N- and C-terminal truncations centered around PA3 peptide were created, and ascertained the minimum binding for 14H6’s epitope as aa 21–30 (bottom panel). **(B)** Amino acid sequence conservation analysis of HPV-L2. Using Weblogo, the 14H6 mAb epitope sequence logo was created with 116 types of HPV L2s (http://weblogo.berkeley.edu/logo.cgi). Sequence data were taken from the NCBI database. **(C)** Sequence comparison of aa 14–36 among various genotypes of HPV L2 protein. Residues that differed to those of HPV16 L2 are indicated by color differences. The region containing the 14H6 mAb epitope is boxed. **(D)** Alanine scanning mutagenesis of the epitope of mAb 14H6. Mutant C22A, K23A, T27A, C28A and P29A abrogated the binding reactivity of mAb 14H6, indicating that these aa were involved in L2-14H6 interaction. **(E)** Reactivity of 14H6’s epitope of HPV 59, HPV31 and HPV6 L2 with respect to HPV16. HPV59 and HPV31 demonstrated lower reactivity, which is linked with their sequence divergence from HPV16. Data are the mean ± SEM.

We first used western blotting with N-terminal (aa 1–265) segments of HPV L2 to identify 14H6’s epitope, and found it located on the L2 N-terminus. In primary screening, an aa stretch (aa 1 to 265) of HPV16-L2 was equally divided into four overlapping 65-mer peptides (PA, PB, PC, and PD) to ascertain where mAb 14H6 interacts. The mAb 14H6 only reacted with PA of HPV L2, corresponding to aa 1 to 65 (Fig 2A, upper panel). In a secondary screening, we synthesized seven overlapping 15-mer peptides spanning aa 1 to 75 (PA1, PA2, PA3, PA4, PA5, PA6, and PA7), and found positive activity with PA3 using ELISA, thus further narrowing down the location of 14H6’s epitope to be within aa 21 to 35. Finally, we used progressive, single aa reduction from both ends of PA3, generating twelve fragments (P A3-1, P A3-2, …, and P A3-12) and found that peptides beyond aa 21 at the N-terminus or without aa 30 at the C-terminus lost reactivity with mAb 14H6, thus identifying a strip of aa 21 to 30 with sequence of TCKQAGTCPP as 14H6’s epitope (Fig 2A, lower panel).

Sequence alignment of this aa stretch among genotypes was performed using MEGA 5.05 and Weblogo programs, and the results showed that aa 24 and 25 are highly varied among HPV subtypes (Fig 2B and 2C). Furthermore, alanine scanning mutagenesis and subsequent ELISA demonstrated that mutation of C22A, K23A, T27A, C28A or P29A abrogated the binding between 14H6 mAb and L2, and mutation of T21A or P30A decreased the binding (Fig 2D), indicating that these residues are involved in the interaction between L2 and mAb 14H6. Campos et al. [32] has reported that C22 and C28 are essential for papillomavirus infectivity, which is consistent with our findings. In addition, Rubio et al. [21] found that aa 30 (serine for HPV31 and HPV59, but proline for other types) plays an essential role in the interaction of HPV31 L2 and mAb K4L2 (epitope of aa 20–38), with mAb K4L2 presenting a lower neutralization for HPV31 PsV as compared with the others tested. Similarly, our mAb 14H6 also discriminates between P30 and S30 of 14H6 peptide reactivity for HPV types (Fig 2E). Overall, the conservation of most of the residues from 14H6’s epitope among HPV genotypes may imply that the epitope has a broad neutralization capacity, which should be exploited for the development of a pan-HPV vaccine immunogen.

### Chimeric constructs of 14H6’s epitope grafted to HPV16-L1, HBc149 and CRM197/389/A scaffold protein

**Fig 3:**
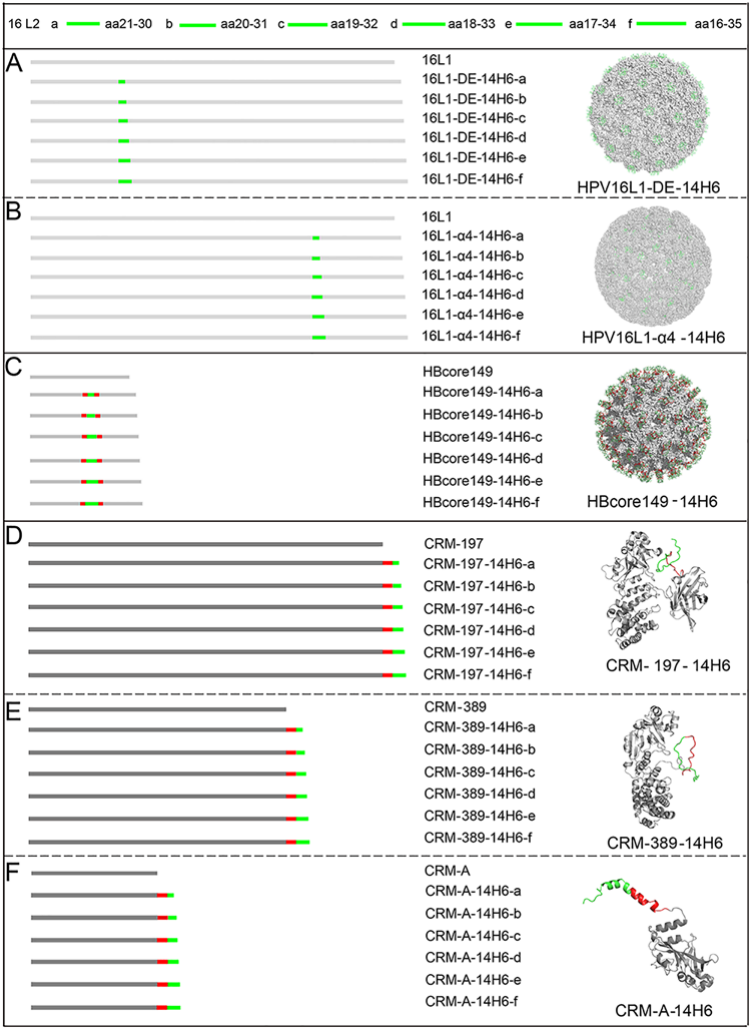
Structural model of 14H6a-f grafted onto HPV16-L1, HBcore149 VLPs, and CRM 197/389/A. Six 14H6 epitope peptides (14H6a–f) (colored in green) were designed to be grafted to: **(A)** the DE loop (130/131) of HPV16-L1; **(B)** the α4 loop (136/137) of HPV16-L1; (C) the major immune-dominant region (MIR; 79/83) of HBcore149; and **(D, E, F)** the C-termini of CRM197, CRM389 and CRMA, respectively. The moieties in red depict the flexible aa stretches flanking both sides of the L2 inserts in the HBcore149 and following the C-terminus of CRM197, CRM389 and CRMA, which are included for optimal surface display. Homology structural modeling of the recombinant VLPs and proteins was implemented using MODELER module of Accelrys Discovery Studio 2.5. All illustrative models were prepared using PyMol [51].

Using PA3-11 as a core region, we next designed six HPV16-L2 14H6 peptides, 14H6a–f, for insertion into the scaffold proteins (Fig 3). Centering on aa 21 to 30, each peptide became progressively longer with the addition of an aa to each end; it was hypothesized that such extensions might allow for the better exposure of the peptide epitope on the scaffold proteins. It was previously shown that there is a limited insertion capacity for maintaining VLP assembly in the HPV16-L1-α4 loop [33]. Therefore, prior to generating our constructs, we used Accelrys Discovery Studio software (San Diego, CA) to visualize and predict the spatial position of the heterologous insert on the homology-modeled structures of the 14H6 chimeras (Fig 3). 14H6a–f fragments were grafted into position at aa 130 in the DE loop and aa 426 in the α4 loop of HPV16 L1; the major immune-dominant region (MIR) (79/83) of HBc149; and directly following the C-terminus of CRM197, CRM389, and CRMA. These built structural models suggested that the inserted peptides would be fully exposed under thermodynamically favorable condition and, therefore, would probably present L2-specific antigenicity and immunogenicity upon immunization.

**Fig 4:**
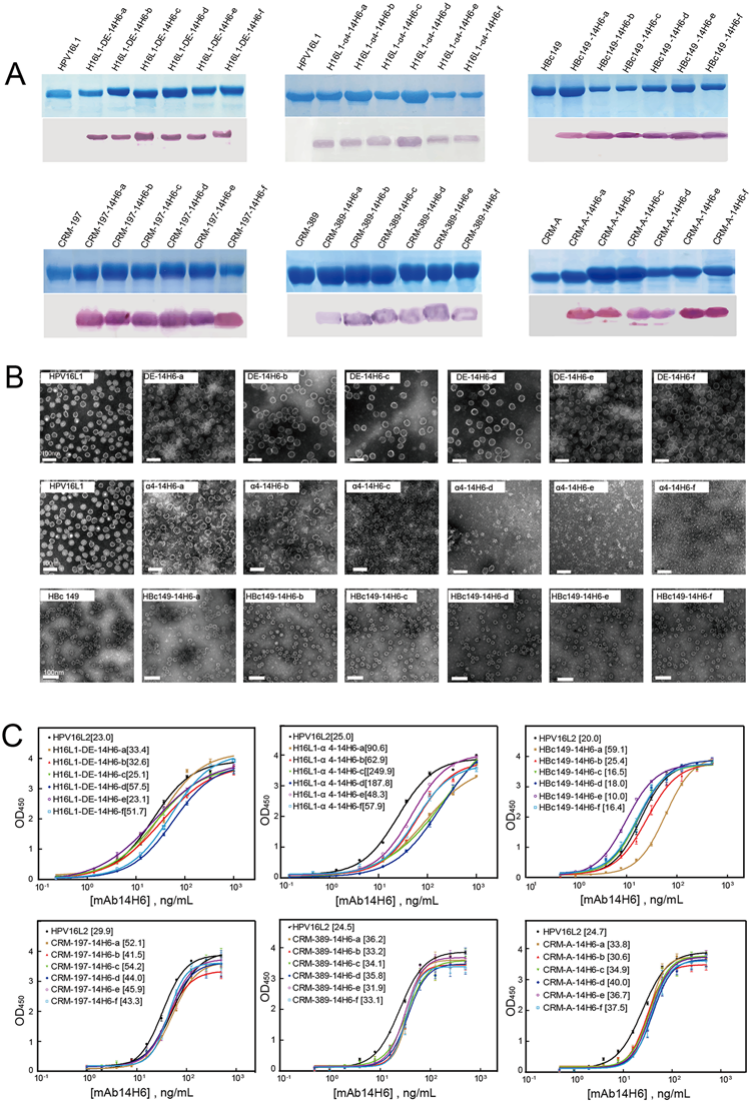
Characterization of 14H6a–f chimeras. **(A)** SDS-PAGE and western blotting. **(B)** TEM visualization for the recombinant protein in virus-like particle (VLP) form. **(C)** EC50 test by ELISA using a serial dilution of mAb 14H6. The reaction curves vs. the concentration of mAb 14H6 were fitted to a sigmoid trend and the EC50 values were calculated in terms of the half points (shown in the brackets). Detection in western blotting and the EC50 results indicate that all chimeric proteins present mAb 14H6 with similar binding capabilities.

All of the 14H6a–f chimeras were expressed in *E*. *coli* and purified to considerably highly purity, as shown in SDS-PAGE and 14H6-binding western blotting (Fig 4A). The chimeras with potential VLP forms were visualized in negative stain transmission electron microscopy (TEM), which revealed that all six HPV16-L1-DE-14H6a–f proteins were able to form uniform VLPs (Fig 4B). Particles with an average diameter of 50 nm were observed, similar to that of wild-type VLPs. However, for the α4 loop chimeras, HPV16-L1-α4-14H6a–f, we noticed a declining trend in particle size and conformity concomitant with an increase in peptide length (Fig 4B). HPV16L1-α4-14H6a, and-b formed VLPs with a mean diameter of 50 nm, whereas HPV16L1-α4-14H6c VLPs were notably smaller with an approximate mean diameter of 30 nm. Large irregular aggregates of pentameric capsomeres were observed for HPV16L1-α4-14H6d, -e, and—f, all of which failed to form uniform VLPs (Fig 4B). On the other hand, the morphologies of the six HBc149-14H6a–f chimeras were similar to that of wild-type, with healthy globular particles of a mean diameter of 30 nm (Fig 4B). Therefore, the HPV16-L1-α4 loop, the HPV16-L1-DE-loop and MIR of HBc149 can harbor at least 20 aa while still maintaining a self-assembling capacity.

Finally, the 14H6-specific antigenicities of the purified chimeras were evaluated by direct ELISA. In general, the fusion proteins and VLPs exhibited similar EC50s to those of the positive controls, with the exception of the HPV16L1-α4-14H6a–f VLPs (Fig 4C). The reactivity of HPV16L1-α4-14H6a–f VLPs, most significantly, VLPs of HPV16L1-α4-14H6c, were notably reduced, as indicated by the increased EC50; this was perhaps due to the structural instability of the recombinant complexes as perceived in the TEM.

### Immunogenicity of 14H6a-f Chimera

**Fig 5:**
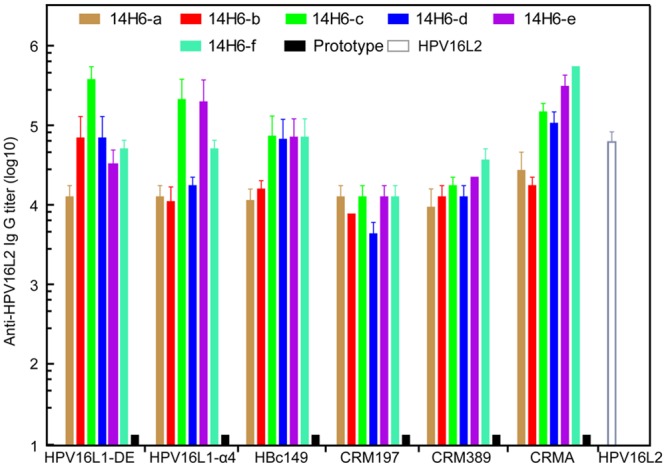
Total L2-specific IgG titer in mice elicited by 14H6a-f chimeras. Chimeric purified proteins of 14H6a–f and scaffolding proteins were inoculated into mice to investigate the specific immunogenicity of 14H6’s epitope. Prototype scaffold proteins and HPV16 L2 served as negative and positive controls, respectively. The antibody level was detected by HPV16 L2-coating ELISA. Significantly, all of the chimeras induced L2-specific responses that were comparable with the HPV16 L2 control. In the case of the CRM chimeras, the A domain fused with 14H6c–f, thereby eliciting a markedly higher antibody titer, similar to that of the other scaffolds (HPV VLP or HBc antigen).

BALB/c mice were vaccinated three times at two-week intervals with 5 μg of the 14H6a–f recombinants absorbed with aluminum hydroxide adjuvant to confirm an anti-HPV16-L2 IgG response. The antibodies were then titered via ELISA. The anti-HPV16-L2 IgG was detectable as early as two weeks after the first immunization (data not shown). By the end of week 8, IgG responses were at maximum levels. With the exception of CRM197 and CRM389 recombinants, at least one of the 14H6 recombinants for each vector elicited a 10^5^ IgG response (Fig 5).

**Fig 6:**
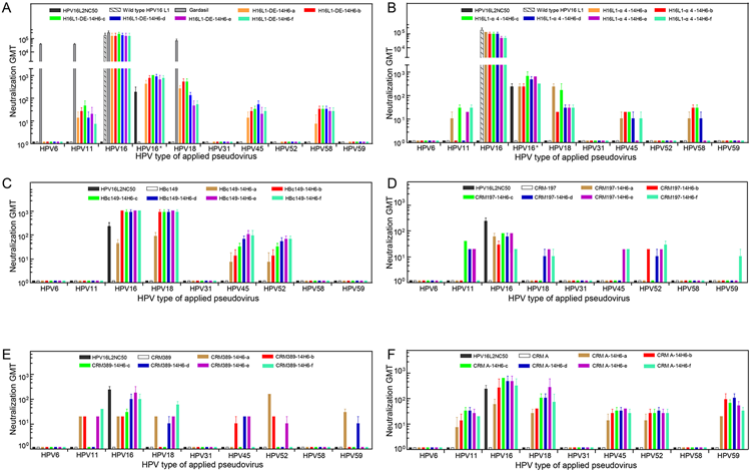
Cross-neutralization titer in mice elicited by 14H6a-f chimeras. Immune sera were titrated using a pseudovirion-based neutralization assay. A total of nine HPV L2 genotypes of pseudoviruses (PsVs) were found: HPV6, 11, 16, 18, 31, 45, 52, 58 and 59. All PsVs were produced in 293FT cells with the co-transfection of homogenous L2 and L1, with the exception of that for HPV16 L2, where HPV16 L2 gene and HPV52 L1 gene were co-transfected to produce a novel PsV, which may eliminate the interference seen with the HPV16 L1 neutralizing antibody. The anti-HPV6/11/16/18 neutralization titers of Gardasil as positive control are shown in gray. Neutralization geometric mean titers (GMTs) were determined for 14H6a–f grafted to **(A)** DE loop of HPV16 L1; **(B)** α4 loop of HPV16 L1; **(C)** the major immune-dominant region (MIR) of HBc149 and **(D, E, F)** the C-termini of CRM197, CRM389, and CRMA, respectively. HPV16*, type-hybrid pseudovirion made of HPV52 L1 and HPV16 L2 used to measure HPV16 L2-specific neutralization titer induced by the constructs of 14H6-epitope embedded into HPV16 L1 VLP.

To determine and compare the immunogenicity and broad neutralization activities of each recombinant 14H6a–f protein and VLP against HPV types 6, 11, 16, 18, 31, 45, 52, 58 and 59, subsequent neutralization assays were performed using the antisera from week 8 (Fig 6). Significant cross-neutralizing activities were detected for the 14H6 peptide displaying HBc149 VLPs, HPV16-DE-L1 VLPs and CRMAs. Inoculation of HBc-149-14H6a–f VLPs prompted immunity against HPV16, 18, 45 and 52 PsVs. The neutralizing geometric mean titer (GMT) was exclusively high against HPV18 PsV at 10^3^, which was equivalent to that against HPV16 PsV (Fig 6C). A comparative study demonstrated stable and robust neutralizing activities of the HPV16-DE-14H6a–f VLP-induced antibodies as compared with that of the HPV16-α4-14H6a-f VLPs, which is concordant with the results from the ELISA and TEM assays (Fig 6A and 6B). Moreover, the E.coli-derived wild-type HPV16 L1 protein and its 14H6 L2-chimeras manifest their excellent L1-specific immunogenicity as being comparable with HPV16/18/6/11 quadrivalent vaccine (Gardasil). However, the HPV16 L2-specific titer is 2-log lower otherwise broader-genotype than L1-specific (see HPV16* assay of chimeras vs. HPV16 assay of HPV16 L1 VLP), which makes the difference of neutralization titers between L1 alone and L1+L2 undiscernible (Fig 6A). Interestingly, we observed a difference in the titers of the cross-neutralizing antibodies produced from CRM197/389/A-14H6a–f (Fig 6D, 6E and 6F): CRMA provoked a greater and broader cross-type neutralizing reaction compared with CRM197 and CRM389, implying that the catalytic domain A of DT alone was superior for presenting the heterologous L2 peptides. Taken together, we show that effective positioning of 14H6 peptides on various scaffold vectors, particularly CRMA, upon immunization elicits the production of broad, cross-neutralizing antibodies against at least HPV16, 11, 18, 45, 52, 58, 59 PsVs. These data provide important insight into an advantageous strategy for the development of a broad-spectrum pan-HPV vaccine.

## Discussion

In this study, we obtained an anti-HPV16-L2 neutralizing antibody, 14H6, that also cross-neutralizes HPV6, 11, 16, 18, 31, 33, 35, 45, 52, 58, and 59 PsVs. It has hitherto one of the widest ranges of cross-neutralization and displays a remarkable neutralizing GMT for each targeted HPV (Table 2). The 14H6 epitope was then mapped through the use of overlapping synthetic peptides, and found to recognize a linear stretch at L2 aa 21 to 30 near its N-terminus. Previous studies have revealed that N-terminal regions of L2 confer multiple, key, functional roles that are essential for virus attachment and viral genome assembly. These regions include DNA binding domains (aa 1–12) [34], cyclophilin B and β-actin binding domains (aa 25–45) [35], and a furin cleavage site (aa 9–12) [18]. During virus adhesion, furin proteases on the cell surface cleave the L2 N-terminus to expose aa 13 to 31 [36]. It was proposed that, since this exposed sequence triggers a critical interaction with a putative L2 receptor to allow virus entry, it must be highly conserved.

**Table 2:** Summary of Anti-HPV L2 Epitope Immunizations.

mAbs	Cross-neutralization against HPV Types	Epitope Location	Identification Methods	Immunogen Designed for Immunogenicity Assay	HPV Types Detectable in Neutralization Assay	References
14H6	HPV6/11/16/18/31/33/35/45/52/58/59	16L2 aa 20–31	synthetic peptides, Alanine-scanning	L2 peptide displayed on HPV16 L1 VLP (α4 /DE loop)	11/16/18/45/58	this work
				L2 peptide displayed on HBc 149 VLP (MIR)	16/18/45/52	this work
				L2 peptide displayed on CRM 197/389/A (C-terminus)	11/16/18/45/52/59	this work
RG1	HPV16/18	16L2 aa 17–36	synthetic peptides	Tandem repeats of L2 peptide displayed on IgG1 Fc	5/6/11/16/18/45/52/58	[20,23]
				L2 peptide displayed on HPV16 L1 (414 aa.)	16	[20,52]
				L2 peptide displayed on PP7 VLP (AB loop)	16/31/45	[20,53]
				Bivalent HPV 16&31 L2 peptides displayed on AAVLP (VP3 453/587 aa.)	16/18/31/45/52/58	[20,24]
				L2 peptide displayed on BPV1 L1 (DE loop)	5/11/16/18/45/52/58	[14,20]
K4L2, K18L2	HPV16/18/27/45/58/57	16L2 aa 20–38	synthetic peptides	N/A	N/A	[21]
mAb 13B, mAb 24b	HPV16/18, HPV16/18/31/33/51/58	16L2 aa 64–73, 16L2 aa 58–64	synthetic peptides	N/A	N/A	[54]
mAb5, mAb13	HPV6/16	16L2 aa 108–120	synthetic peptides, Alanine-scanning	L2 peptide displayed on HPV16 L1 (414 aa.)	HPV16	[52,55]
				L2 peptide displayed on BPV1 L1 (DE loop)	HPV11/16	[55,56]
N/A		16L2 aa 11–88		Concatenation of 8 HPV types L2 peptides displayed on flagellin	HPV16/33/35/56	[57]
				Multivalent protein comprised of concatenation of 8 HPV types L2 peptides	HPV6/16/26/31/33/35/45/51/56/58/59	[58]
				Multivalent protein comprised of concatenation of 8 HPV types L2 peptides co-immunize with Gardasil or Cervarix	HPV16/18/35/45/58	[17]
				Multivalent protein comprised of concatenation of 11 HPV types L2 peptides	HPV16/18/45/58	[59]

HPV, Human Papillomavirus; mAb, monoclonal antibody; MIR, major immune-dominant region; VLP, virus-like particle.

Several HPV16-L2-based cross-neutralizing antibodies have been identified, and these epitopes either envelope or overlap with that of 14H6 (Table 2). For example, RG-1 by Gambhira et al. and K4L2_20-38_ by Rubio et al. target aa 17 to 36 and aa 20 to 38, respectively [20,21]. Whereas mAb RG-1 displayed a neutralization titer of 1280 against HPV16 and 18 PsVs, K4L220-38 had a neutralizing titer ranging from 625 to 3125 against HPV16, 18, 45, 58, 57, and 27 PsVs. Comparatively, our mAb 14H6 appears to exhibit a much higher and wider cross-neutralizing activity, even though it shares a similar neutralization profile with these previously described mAbs; albeit, the neutralization against HPV31 and HPV6 are lower.

Immunization of L2 oligopeptides have previously shown to induce relatively low neutralizing antibody titers [37]; this is despite efforts to modify the aa sequence through the use of tandem repeats of an L2 peptide on bacterial thioredoxin or concatenating L2 peptides of different papillomavirus types, among others (Table 2). Immunologically, the unique display of certain peptides, such as in the form of VLPs, intra-molecular adjuvants, and as oligomeric proteins, have been able to enhance the immunogenicity of the intended epitope polypeptides; in VLPs or oligomers, the immunogens can carry multivalent peptides to cover variants among a target sequence. For more than 20 years, HPV VLPs and HBc149 VLPs have been frequently used as epitope-display particle vectors [38,39]. In our laboratory, we have frequently used insertions of diverse short peptides into one of the two constrained surface-exposed loops—the DE-loop (130/131) or the α4 loop (426/427)—on the HPV16-L1 protein, and found that such insertions do not affect the L1’s ability to self-assemble into immunogenic VLPs (data not shown). A C-terminal truncations of HBc149 has also been widely used as immune-enhancing carrier proteins [40]. Upon the formation of VLPs, an abundance of peptide inserts are presented on the surface as foreign antigens [41].

Our data and previous studies are in agreement that the DE loop of HPV L1 is better at peptide display than the α4 loop, since it is able to accommodate inserts with more than 30 aa while maintaining high immunogenicity [14,33]. This assertion was supported by findings from atomic modeling of the bovine papillomavirus (BPV), which showed that, although the α4 loop is exposed on the surface of VLPs, it also serves an important function in VLP assembly. Upon insertion of a peptide with more than 16 aa, the α4 loop will distort, and this may lead to assembly failure of the neighboring pentameric capsomeres; indeed, discrete aggregates and pentamers were observed in this study under TEM. Our study has also demonstrated that insertion of 14H6a–f into the MIR of HBc149 did not affect the assembly of the HBcore VLPs, and was able to elicit a remarkable cross-neutralizing response against HPV16, 18, 45, and 52. In addition, the 14H6 CRMA chimeras exhibited a wider spectrum and an equivalent cross-neutralizing potency of six HPV types (HPV11, 16, 18, 45, 52 and 59) as compared with the 14H6 HPV16-L1-DE and HBcore VLP chimeras. Furthermore, fusion of CRMA with the 14H6 epitope was more effective in terms of peptide display than CRM197 and CRM389, suggesting that the catalytic domain A of CRM197 may favor peptide folding and be potent in immunogenic enhancement. It should be mentioned here that this is the first time CRMA has been exploited and shown to be a more effective display vector than the other moieties and intact CRM197.

In our immunogenicity analysis using multiple HPV types for neutralization, none of the 14H6 chimeras could induce marked cross-neutralization titers in anti-14H6 sera, despite CRMA-14H6 showing neutralization for six HPV types (Fig 6). However, in the neutralization assay, mAb 14H6 was shown to exhibit high and wide-genotype neutralizing capacity (Fig 1C). This discrepancy reflects the persistent challenge associated with evoking a host immune response in an immunodominant manner against a linear epitope. During vaccination, different scaffold proteins may contribute to changes in peptide folding during recombinant expression and subsequent immune presentation. In terms of sequence conservation for the 14H6 epitope, there is still variation among the HPV genotypes. In a word, our findings suggest an L2-based pan-HPV vaccine should cover extensive L2-epitope chimeric constructions to elicit broader HPV type protection coverage, at least including scaffold protein fusion design, peptide length variation and HPV L2 sequence diversity. In future experiments, the 14H6 epitope peptide should be instead designed using key residues from a common motif, TCKxxxTCPP, in order to cover more of the HPV genotypes. This modified peptide should then be grafted to CRMA and/or HPV VLPs to confer considerable immunogenicity. This would indeed help in the generation of an L2-based, pan-HPV vaccine, with future L2-based vaccine clinical trials revealing the neutralization mechanism of L2 relative to that of the known L1.

## Materials and Methods

### Ethics Statement

In this study, the experimental animals (BALB/c mice) were purchased from Shanghai Institutes for Biological Sciences (Shanghai, China), and fed in the animal facility of Xiamen University Laboratory Animal Center (XMULAC). The manipulation and vaccination on the animals were strictly referred to the guideline and compliant with the regulation, which was provided by XMULAC. Prior to the implementation, the experiment schemes and protocols were reviewed by Xiamen University Institutional Committee for the Care and Use of Laboratory Animals and approved by Xiamen University Laboratory Animal Management Ethics Committee. During the experiments, all animals were well-fed and monitored twice per day. The manipulation, including vaccination, blood collection and surgery, was handled by well-trained professional in terms of the essential principle of gentle and painless. Finally, experimental mice were injected intraperitoneally with nembutal sodium for euthanasia.

### HPV16 C50L2 and GST fusion peptides

The gene encoding HPV16 C50 L2 (aa 1–423, GenBank no. KF181716.1) was cloned into pTO-T7 vector [42] for non-fusion expression in *E*. *coli* ER2566. The genes encoding the HPV 16L2 GST fusion peptides—PA (aa 1–65), PB (aa 50–115), PC (aa 100–165), and PD (aa 150–215)—were individually cloned into the PGEX-20T vector for *E*. *coli* expression. The target protein and fusion peptides were then purified from the inclusion body to >90% purity using methods previously described [43,44]. A bicinchoninic acid protein assay was performed to determine the concentration of the purified HPV16 L2 proteins.

### Murine monoclonal antibodies (mAbs)

BALB/c mice were immunized subcutaneously three times at an interval of two weeks with HPV16C50L2 (20 μg/animal) absorbed with Freund’s Complete Adjuvant. The anti-HPV16L2 murine mAbs 14H6, 15E5, 15E4, and 6H8 were then produced using the hybridoma technology previously described [45]. Protein A affinity chromatography was employed to purify the anti-HPV mAbs IgGs and the purified mAbs were subsequently diluted to 1.0 mg/ml in PBS and stored at −20°C.

### Oligopeptide generation

Peptides were synthesized using the Fmoc method outlined by GLS (GL Biochem, Shanghai, China). Seven overlapping 15-mer peptides (PA1, PA2, PA3, PA4, PA5, PA6 and PA7) were designed across HPV16-L2 aa 1 to 75. From the competitive ELISA test, PA3 was the only 14H6-reactive peptide; thus, PA3 was then further truncated at the N-terminus (PA3-1, PA3-2, PA3-3, PA3-4, PA3-5, PA3-6) and C-terminus (PA3-7, PA3-8, PA3-9, PA3-10, PA3-11, PA3-12). The sequences of these polypeptides are shown in Fig 2B.

### Homology modeling

Using protein BLAST search (NCBI, www.ncbi.nlm.nih.gov/blast/), three crystal structures extracted from Protein Data Bank (PDB no. 1DZL, 1QGT and 4AEO matching HPV16L1, HBc149 and CRM, respectively) were selected as templates for homology modeling of the corresponding monomers. After sequence alignment with the respective 14H6 chimeric sequences, initial 3D models were generated using the Homology module of Discovery Studio 2.5 program (Accelrys). BPV (PDB no.3IYJ) and HBc149 (PDB no. 1QGT) VLP templates were used to generate the complete HPV16L1-14H6 and HBc149-14H6 VLP models. Stepwise minimizations were subsequently applied to the 14H6 insertion site of each model to produce thermodynamically favored conformations.

### Construction of 14H6 chimeras

The 14H6 epitope, located in the N-terminus of HPV16 L2 from aa 21 to 30, has a decapeptide sequence (TCKQAGTCPP). For adequate surface exposure of the 14H6 epitope on the fusion VLP and proteins, 14H6 (14H6a), was modified in six different ways by extending either or both ends to form 14H6-b, a dodecapeptide (aa 20–31), 14H6-c, a tetradecapeptide (aa 19–32), 14H6-d, a hexadecapeptide (aa 18–33), 14H6-e, an octadecapeptide (aa 17–34), and 14H6-f, an icosapeptide (aa 16–35). DNA encoding of the peptides was undertaken using recombinant mutagenesis following a two-step PCR protocol, described elsewhere [46]. HPV16 L2 14H6-a–f constructs were subsequently cloned into the DE loop (130/131) and α4 loop (426/427) of HPV16 L1, the MIR (79–83) of HBV core protein (HBcAg), and directly after the C-terminus of CRM197, CRM389, and CRMA; all of the genes encoding scaffold proteins were cloned into pTO-T7 plasmids. In addition, a 15-aa flexible peptide linker (GGGGSGGGGSGGGGS) was introduced to the C-terminus of the inserted epitope in the CRM197, CRM389, and CRMA constructs to allow for its potential surface presentation. For the HBV core, a 10-aa flexible peptide linker (GGGGSGGGGS) was added in duplicate to flank both sides of the inserts.

### Expression and purification of 14H6 chimeras

The genes encoding HPV16 L1-14H6, HBc149-14H6 and CRM-14H6 chimeras were cloned into pTO-T7 vector[42], and transformed to *E*. *coli* ER2566 for chimeric expression. The transformated bacteria were cultured in LB medium at 37°C overnight and the protein expression was initiated by the addition of isopropyl-β-D-thiogalactoside (IPTG, final concentration of 10μM) and carried out with further incubation at 25°C for 6 h. Cells were harvested by centrifugation and resuspended with cell lysis solution (20 mM Tris, pH7.4, 300 mM NaCl, and 5 mM EDTA). The 14H6 chimeras were released from the cells by sonication and separated from the cell debris by centrifugation. As for HPV16 L1-14H6 and CRM -14H6 chimeras, the interest proteins in lysate supernatants were precipitated by ammonium sulfate of 25% saturation while incubating at 4°C for 2h. The precipitates were resuspended in 20mM PB pH 8.0, 500mM NaCl. Then, HPV16 L1-14H6 proteins with the dilution of equal volume 20mM PB, pH 8.0, 40mM dithiothreitol (DTT), were further purified by cation ion exchange chromatography (SP Sepharose 6FF resin, GE Healthcare, Uppsala, Sweden) and eluted in 800 mM NaCl fraction; and CRM -14H6 proteins with final NaCl concentration of 2.0 M were further purified by hydrophobic interaction chromatography (Phenyl Sepharose 6FF resin, GE Healthcare) and eluted with 200mM NaCl. For HBc149-14H6 chimeras, the lysate supernatant were treated with heating at 56°C for 30min to eliminate most contaminants, then the resultant supernatant was precipitated by 40% saturated ammonium sulfate. Precipitates were resuspended with PBS (pH 7.4) and further purified with a DEAE-5PW column (TOSOH Bioscience, Tokyo, JP). Finally, the purified HPV16 L1-14H6 chimeras self-assembled into virus-like particles with the removal of reductant DTT.

### SDS-PAGE and western blotting

The 14H6 chimeras were analyzed by SDS-PAGE using methods outlined by Laemmli with slight modifications. Protein samples were mixed with equal volumes of 6× loading buffer (composed of 100 mM Tris-HCl, pH 6.8, 200 mM BME, 4% SDS, 0.2% Bromophenol blue and 20% glycerol). Sample mixtures were heated at 80°C for 10 min and subsequently loaded onto 10% acrylamide gels. For western blotting analyses, separated samples of the 14H6 chimeras were transferred from the SDS gels to nitrocellulose membranes. The membranes were blocked with 5% skim milk, soaked in 1:500 diluted HPV16 L2-specific mice sera, and incubated at room temperature for 1 h. Subsequent washing was performed using 0.2% Tween-20 in phosphate-buffered saline, pH 7.4. Alkaline phosphatase-conjugated secondary antibody (Dako, Denmark) was then added to capture the bound primary antibody. A mixture of nitro blue tetrazolium and 5-bromo-4-chloro-3-indolyl phosphate was then added to allow color development and visualization of the target protein bands.

### Transmission electron microscopy (TEM)

Approximately 15 μl of negatively stained HPV16L1- and HBc149-vectored 14H6 chimeric VLPs at 200 μg/ml were absorbed onto carbon-coated copper grids, blotted dry, and stained with freshly filtered 2% phosphotungstic acid (pH 6.4). Grids were examined under the FEI Tecnai T12 TEM at an accelerating voltage of 120 kV and then photographed at a nominal 25,000× magnification.

### Direct binding ELISA

14H6 chimeric proteins (300 ng/well) were coated into the wells of 96-well microplates. After plate blocking, 100 μl of 2-fold serially diluted anti-HPV16L2 mAbs 14H6 (initiated from 1 μg/ml in PBS) were added to the wells. HRP-conjugated goat anti-mouse Ig antibody (diluted 1:5000 in HS-PBS, Abcam; Cambridge, UK) was used as a secondary antibody. The absorbance at 450 nm with reference to 620 nm was record using an automated ELISA reader (TECAN, Männedorf, Switzerland). The cut-off value was set as absorbance of 450 (Δ620)nm = 0.2 to define the positive titer.

### Animal immunization

Four-to-six weeks old female BALB/c mice were divided into forty-two groups (n = 4 in each group) and each group of mice was vaccinated subcutaneously three times, each separated by an interval of two weeks. 5 μg of immunogens, (14H6 chimeric proteins and negative controls including HPV16C50L2, HPV16 VLP, HBc149, CRM197/389/A) absorbed with aluminum hydroxide adjuvant and 1/8 human dose of HPV16/18/6/11 quadrivalent vaccine (Gardasil) were injected subcutaneously on day 0, 14 and 28. Serum samples were collected individually from each mouse at the 14th day after each injection. Serum samples on the fifty-sixth day were used to analyze the neutralizing titers by following neutralization assay. The neutralization titers of 4 mice per group were plotted in averaged value with standard error vs. various types of applied HPV pseudoviron.

### Pseudovirus neutralization assay

HPV6, 11, 16, 18, 31, 33, 45, 52, 58 and 59 pseudo-viruses were produced according to previous studies [37,47,48]. For the specificity for HPV16 L2 neutralization assay, HPV16 L2 pseudovirus (denoted as HPV16*) was generated with the genes of HPV52 L1 and HPV16 L2 to eliminate the interference of HPV16 L1 neutralizing antibody, which may be produced by HPV16 L1 VLP as scaffold protein. The L1/L2 expression vector and pN31-EGFP used in the experiment were kindly provided by Dr. J. T. Schiller [49]. 293FT cells were harvested 72 h after transfection, lysed with cell lysis buffer 0.5% Brij58 (Sigma-Aldrich; St Louis, MO), 0.2% Benzonase (Merck Millipore; Darmstadt, Germany), 0.2% PlasmidSafe ATP-Dependent DNase (Epicenter Biotechnologies, Madison, WI) DPBS-Mg solution, and incubated at 37°C for 24 h. Afterwards, 5 M NaCl solution was added to the samples to extract the cell lysates. TCID50 (tissue culture infective dose) of the supernatant was then measured to determine the titers of the PsVs, and the TCID50 values were calculated according to the classical Reed—Muench method [50].

### Detection of sera neutralizing antibodies

293FT cells were incubated at 37°C in the wells of a 96-well plate at a density of 1.5×10^4^ cells per well for 6 h. Sera were subjected to a 2-fold dilution. PsVs were diluted to 2×10^5^ TCID50/μl. Sixty μl of the PsV diluent and 60 μl of the serially diluted sera were mixed and incubated at 4°C for 1 h. The negative control was prepared by mixing 60 μl of the PsV diluent with 60 μl of the culture medium. Then, 100 μl of the above mixtures were added designated wells and incubated at 37°C for 72 h. Cells were then treated with trypsin and analyzed by flow cytometry. The endpoint titers were calculated as the log_10_ of the highest sera dilution with a percent infection inhibition higher than 50%. Every sample was run at least three times, and the values presented here are calculated as the mean value of all repeats.
